# Determinants of completion of cancer directed treatment: an experience from a rural cancer centre, Sangrur, Punjab state, India

**DOI:** 10.3332/ecancer.2021.1313

**Published:** 2021-11-01

**Authors:** Atul M Budukh, Debashish Chaudhary, Sankalp Sancheti, Tapas Dora, Alok Kumar Goel, Anshul Singla, Akash Sali, Shraddha Shinde, Kuldeep Singh Chauhan, Prithviraj Kadam, Raza Mohammad, Rakesh Kapoor, Pankaj Chaturvedi, Rajesh P Dikshit, Rajendra A Badwe

**Affiliations:** 1 Tata Memorial Centre (TMC), Homi Bhabha National Institute (HBNI), E Borges Marg, Mumbai, Maharashtra 400 012, India; 2 Homi Bhabha Cancer Hospital (HBCH), Civil Hospital Campus, Sangrur, Punjab 148001, India; 3 Centre for Cancer Epidemiology (CCE), Tata Memorial Centre, Mumbai, India; a https://orcid.org/0000-0001-6723-802X; b https://orcid.org/0000-0001-8917-678X; c https://orcid.org/0000-0001-5810-8733; d https://orcid.org/0000-0001-8316-3435; e https://orcid.org/0000-0003-3789-1591; f https://orcid.org/0000-0003-4830-0486; g https://orcid.org/0000-0002-0480-2831

**Keywords:** cancer care, declining cancer treatment, India, registry, treatment compliance, rural health care

## Abstract

In low and middle-income countries, access to cancer diagnosis and treatment is suboptimal. Further, compliance to cancer treatment is a major issue due to various reasons including financial barriers, lack of family support and fear of treatment. This article discusses the determinants of treatment completion in cancer patients of a government-run hospital, in a rural part of Punjab in India. The Sangrur hospital-based cancer registry data for the year 2018 have been used. We have registered 2,969 cancer cases, out of which 2,528 (85%) cases were eligible for the analysis. Of the total 2,528 cases, 1,362 (54%) cases completed the cancer directed treatment and 1,166 (46%) did not. The data have been collected from the electronic medical record (EMR) department and entered into *CanReg5* software. The bivariate and multivariate binary logistic regression analysis was performed to see the effect of variables on the treatment completion. The results indicate that the elderly age group (>60 years) (odds ratio (OR): 0.52, (95% confidence interval (CI): 0.31–0.86)), distance from hospital (OR: 0.67, (95% CI: 0.50–0.89)) and access to government health schemes (OR: 0.13, (95% CI: 0.10–0.19)] have direct correlation with the treatment completion. The educated patients (OR: 1.49, (95% CI: 1.13–1.96)) and patients who received curative treatment (OR: 2.7, (95% CI: 1.88–3.88)) have shown 58% and 84% compliance to treatment completion, respectively. The other variables like the clinical extent of disease, religion, gender and income do not have any significant effect on the treatment completion. Determinants like age (young), education, distance from the hospital, curative treatment and availability of government health schemes for financial support have shown positive effects on treatment completion. These factors have to be considered by the cancer hospitals, health departments and policymakers while planning for cancer care or control in India.

## Introduction

**Figure 1.**
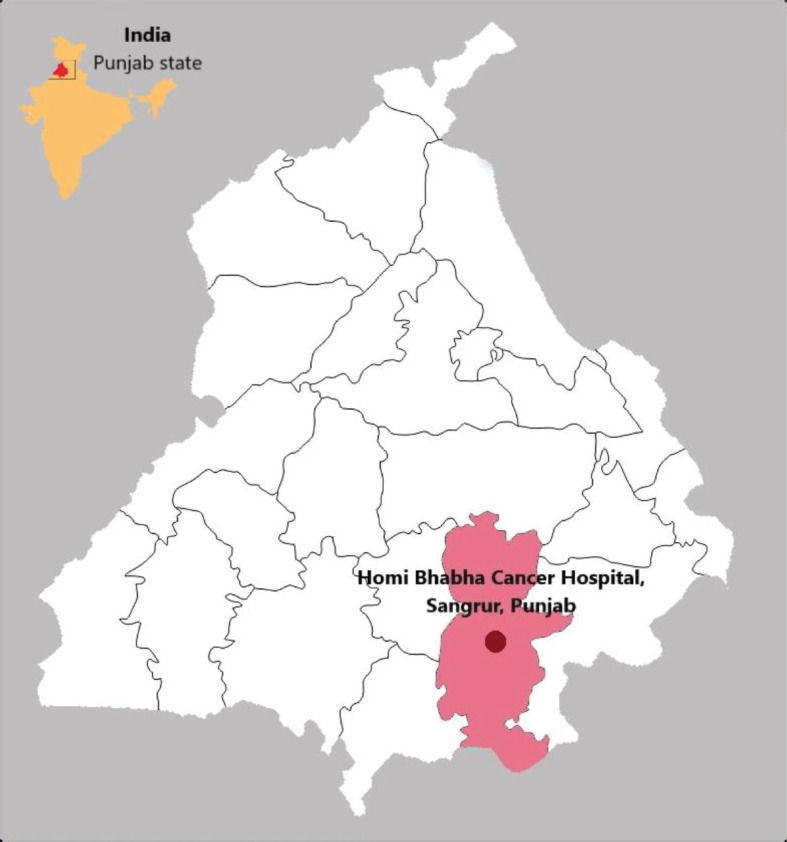
Location of HBCH, Sangrur, Punjab state, India.

Cancer is the leading cause of death globally accounting for 10 million deaths per year. Around one in six deaths is due to cancer [1]. In low and middle-income countries, due to limited access to cancer diagnosis and treatment facilities, most patients are diagnosed with cancer in the advanced stage contributing to low survival rates [2]. Further, the availability of cancer directed treatment is high (90%) in high–income countries compared to low-income countries. As per the hospital-based cancer registries report (2012–2014) of India, the no cancer-directed treatment was in the range of 17%–60% and 15%–50% in males and females, respectively [3]. The percentage of patients who haven’t completed the treatment is high and it has negative effect on the survival rate and quality of life. Several factors such as financial constraints, lack of family support and fear of radiotherapy (RT) treatment affect the treatment completion in cancer patients [4–6]. Moreover, there is no significant difference in cancer mortality in the rural as well as urban areas of India [7]. In this article, we are demonstrating the determinants affecting treatment completion. The data are taken from the hospital-based cancer registry (HBCR) of Homi Bhabha Cancer Hospital (HBCH), Sangrur, Punjab, India. HBCH is established by Tata Memorial Centre (TMC), Mumbai, with the support of Punjab government in the rural area of the Punjab state, which is around 125 km from Chandigarh Union Territory. The location of HBCH Sangrur is depicted in Figure 1.

## Materials and methods

The HBCH, Sangrur is functional since January 2015. The hospital provides holistic diagnostic facilities such as computerised tomography scan, magnetic resonance imaging, ultrasonography, mammography, biochemistry, haematology, tumour marker, histopathology, immunohistochemistry and cytology. Additionally, the hospital provides surgical, RT and medical oncology services based on the treatment protocol provided by TMC, Mumbai. The hospital also provides preventive services in Sangrur like early detection of breast, cervix and oral cancer. This hospital has both population-based and hospital-based cancer registries [8, 9].

The HBCR, Sangrur was established in the year 2017. Two staff members with a science background were selected for cancer case abstraction. Later, these staff received training at Centre for Cancer Epidemiology (CCE), TMC, Mumbai. HBCR proforma was prepared by the CCE unit after a detailed consultation with the HBCH clinicians. The trained cancer registry staff abstracted the information of cancer cases from the EMR under the guidance of the treating consultant. As per hospital system, during patient’s registration at the hospital, the registration clerk records the demographic details like age, sex, education, income, residence and religion of the patient and these records are entered in the EMR system. For illiterate patients, a patient guide (medical social worker) helps in data collection. The registry staff collects these demographic data of EMR from IT department and also gathers cancer cases information such as new or old cases, the clinical extent of disease, health scheme applied and the treatment completion details. As per the registry database, cancer directed treatment is any treatment given to control or destroy the tumour cells and the patient has completed all the assigned treatment; these treatments may include surgery, RT and chemotherapy (CT). Whereas, no-cancer directed treatment is referred for patients who have not received or not accepted the assigned treatment, patients who had incomplete treatment and patients whose treatment status is unknown.

The term ‘new cases’ is referred to patients who have not been to any other hospital for treatment before approaching HBCH Sangrur. While ‘old cases’ are those who were treated initially in other hospitals and visited the HBCH for further treatment.

**Figure 2.**
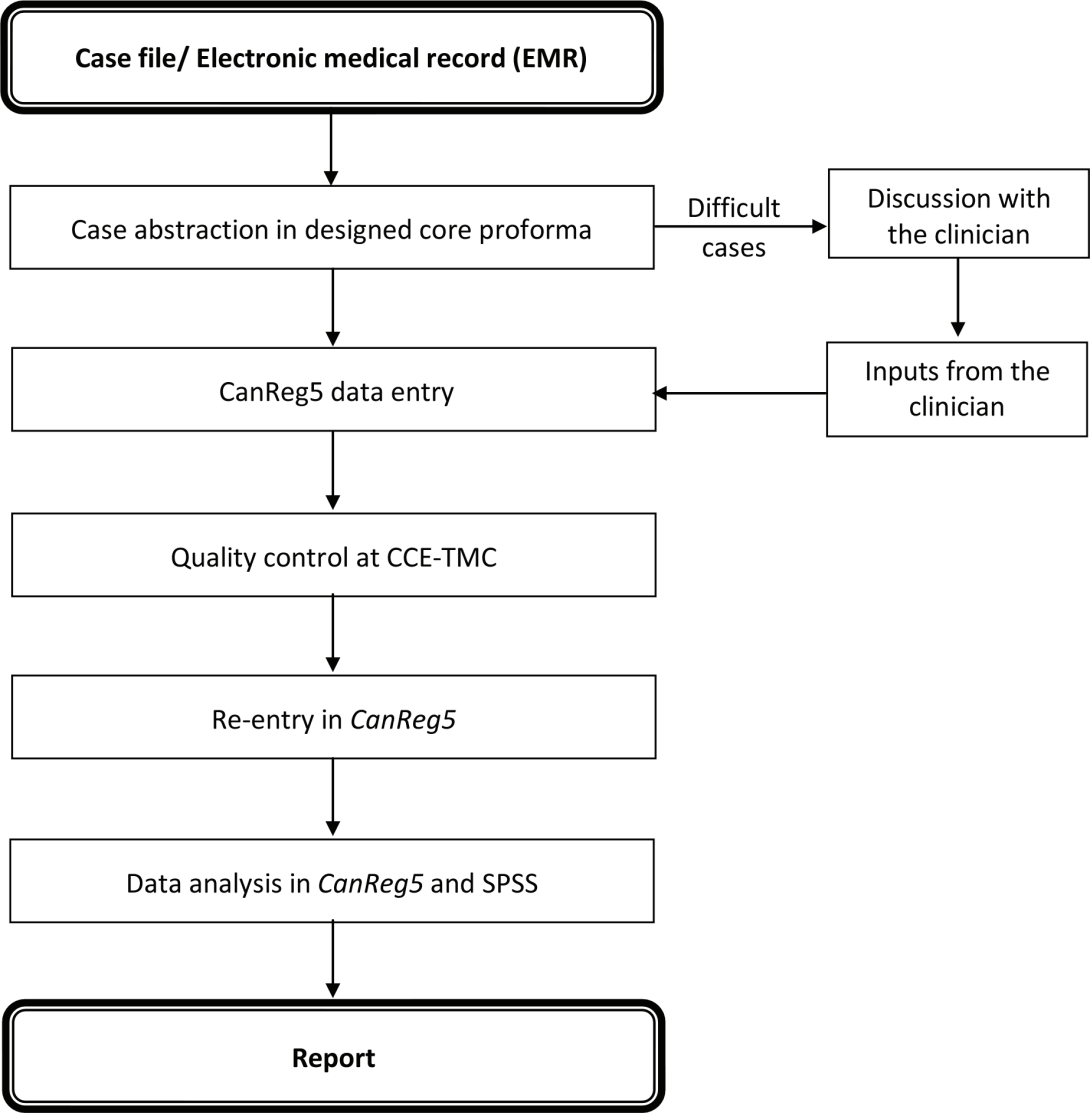
Cancer case registration method.

The primary site and histology are coded using the International Classification of Diseases for Oncology 3rd edition (ICD-O3) [10]. The data of abstracted cancer cases are regularly entered in the *CanReg5* software [11]. This entered data is checked by the senior staff from CCE-TMC for quality control. Any error observed in the case abstraction is discussed with the clinician and registry staff. Moreover, for the accuracy of the data, CCE-TMC staff randomly checked the data through the TMC server and senior staff visited Sangrur to discuss the errors with concerned staff and made sure that the errors were corrected and re-entered carefully into the database. The final data entered is analysed using the *CanReg5* software and *SPSS* software version 21.0 (IBM, Armonk, New York, USA). The cancer registration method is shown in Figure 2.

The data analysis was performed using *Stata* software version 15.0 (StataCorp LLC, College Station, Texas, USA) [12]. Bivariate and multivariate binary logistic regression analysis was used to understand the status of treatment completion in cancer patients at HBCH Sangrur. The treatment completion is referred to patients who have received cancer directed treatment as per the prescribed protocols. The treatment completion is the dependent/binary response variable and the socio-demographic factors (age, gender, income, religion, education, district and state), clinical factors (clinical extent, intent of treatment, type of case) and mode of cost payment for treatment are the set of independent/explanatory variables. The variables which are significant on the univariate analysis have been tested for the multivariate analysis to see the effect on the outcome.

## Results

In the year 2018, we have registered 2,969 cases. After excluding benign, *in situ*, uncertain behaviour cases, the study sample contains 2,528 cases; out of which 1,362 (53.9%) completed the treatment and 1,166 (46.1%) did not. As per socio-demographic data, the majority of the cases are females (54%), around 90% of the total patients are from low income category, most patients are from Sikh community, approximately 56% of patients are illiterate, more than 60% stay out of Sangrur district and around 80% cases are new cases. The detailed socio-demographic characteristics of the cancer patients have been described in Table 1.

**Figure 3.**
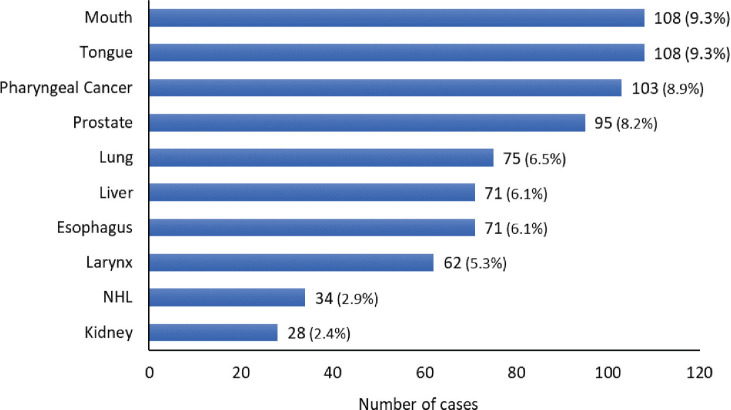
Leading cancer sites at HBCR in males: 2018.

**Figure 4.**
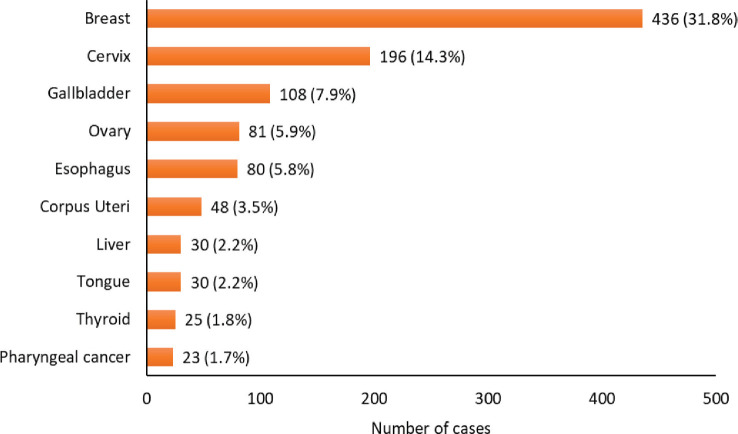
Leading cancer sites at HBCR in females: 2018.

Among males, the predominant cancers are mouth cancer (C03-C06) (108) and tongue cancer (C01-C02) (108) followed by pharyngeal cancers (C09-C10, C12-C14) (103), prostate cancer (95), lung cancer (75), liver cancer (71), oesophageal cancer (71), larynx cancer (62), non-Hodgkin’s lymphoma (34) and kidney cancer (28). The graphical representation of the leading sites in males for the year 2018 is depicted in Figure 3. In females, the predominant cancer is breast cancer (436) followed by cervix uteri cancer (196), gallbladder cancer (108), ovarian cancer (81), oesophagus cancer (80), corpus uteri cancer (48), liver cancer (30), tongue cancer (30), thyroid cancer (25) and pharyngeal cancer (C09-C10, C12-C14) (23). The graphical representation of the leading sites in females in the year 2018 is depicted in Figure 4.

Out of 2,528 cancer cases, 1,362 (53.9%) completed the treatment. Among these 1,362 cases, 126 (5.0%) underwent surgery, 236 (9.3%) CT, 209 (8.3%) RT, 413 (16.3%) RT and CT, 81 (3.2%) surgery and CT, 105 (4.2%) surgery, RT and 192 (7.6%) underwent surgery, RT and CT. The treatment details are presented in Table 2. The univariate and multivariate analysis is presented in Table 3. In the univariate analysis, age, clinical extent of disease, education, district, type of case (old/new), the intention of treatment and payment mode are statistically significant and the gender, income, religion and state are not significant.

In accordance with multivariate analysis, the >60 years age group patients have a lesser chance (odds ratio (OR): 0.52, (95% confidence interval (CI): 0.31–0.86)) of completing the treatment when compared to the <40 years age group and it is statistically significant. It is also observed that 40–59 years age group patients have a lesser chance (OR: 0.63, (95% CI: 0.38–1.03)) to complete the treatment when compared to the <40 years age group, however, it is not statistically significant. Literacy has shown the effect on treatment completion. The educated (literate) patients have a higher chance of completing the treatment (OR: 1.49, (95% CI: 1.13–1.96)) when compared to uneducated (illiterate) patients and it is statistically significant. The place of residence in Sangrur district has shown a positive effect on treatment completion. The other district patients have a lesser chance (OR: 0.67, (95% CI: 0.50–0.89)) of completing the treatment when compared to patients who are residing in the Sangrur district. The effect of residence on treatment completion is statistically significant. The residence status within or out of the Punjab state has not shown any significant effect on the treatment completion.

In the univariate analysis, it was observed that new cases have a higher chance of completing the treatment (OR: 1.23, (95% CI: 1.02–1.50)) as compared to the old cases. However, the multivariate binary logistic regression showed that new cases are having a lesser chance of completing the treatment (OR 0.70 (95% CI: 0.50–0.98)).

In the univariate analysis, it was observed that the cancer cases having extent of disease at the metastasis stage are having a lesser chance of completing the treatment as compared to localised cases (OR: 0.66, (95% CI: 0.53–0.83)]. The cancer cases of unknown stage/not applicable have a lesser chance of completing the treatment as compared to the localised cases (OR: 0.25, (95% CI: 0.19–0.33)). In the multivariate binary logistic regression, due to effect of other variables, there is no statistically significant effect of clinical extent of disease on the treatment completion.

The patient who has undergone curative treatment have higher chance of completing the treatment (OR: 2.7, (95% CI: 1.88–3.88)] compared to a patient who has been given palliative treatment and it is statistically significant.

The hospital is facilitating different government schemes to provide financial help in the course of the treatment completion. The eligible patients who have not applied for the government schemes have a lesser chance (OR: 0.13, (95% CI: 0.10–0.19)) of completing the treatment when compared to those patients who have applied and it is statistically significant. The cancer patients who are spending the money from their own funds for the treatment have a lesser chance (OR: 0.36, (95% CI: 0.25–0.52)) of completing the treatment. Similarly, the government employees who have applied for reimbursement and the patients who applied for other health insurance scheme have a lesser chance of completing the treatment (OR: 0.48, (95% CI: 0.29–0.77)) as compared to the patients who have applied for the government health scheme.

## Discussion

The HBCR Sangrur provided data on treatment completion, status of cancer cases and cancer patients who received no-cancer directed treatment for the year 2018. The treatment completion of all the cancer patients who attended the hospital in the year 2018 is 53.9% (male: 52%; female: 55%) and patients who received no-cancer directed treatment is 46.1% (male: 48%; female: 45%). The no-cancer directed treatment is also reported by other cancer centres in the country such as Tata Memorial Hospital, Mumbai (male: 42.5%; female: 32.9%), Kidwai Cancer Centre, Bangalore (male: 56.5%; female: 47.5%) Cancer Institute Chennai (male: 60%; female: 46.5%) as well as Dr. Bhubaneswar Borooah Cancer Institute (BBCI), Guwahati (male: 50.4%; female: 50.3%) [3].

As the HBCH is located in the Sangrur district, the treatment completion of cancer patients who stays in Sangrur district is high when compared to patients who stay out of Sangrur district. In a systematic review on the distance and cancer treatment, it is reported that cancer patients who have to travel more than 50 miles are usually diagnosed at the advanced stage, they have low adherence to treatment, worse prognosis and poor quality of life. The burden of travel from patient’s residence to health care providers is an important factor that influences the access to diagnosis and treatment [13].

Education (literacy) is an important factor in treatment completion. The educated cancer patients are more likely to complete the treatment. Studies have shown that higher education plays an important role in treatment completion as these patients have the capability to read the cancer treatment related information, visit hospital by their own and effectively communicate with the clinicians. Whereas, uneducated patients mostly depend on family members [14]. Patients with lower health literacy were less likely to receive CT compared with patients with higher health literacy according to a study [15]. It is also reported that low health literacy is associated with diminished screening, advanced stage at diagnosis, decreased acceptance and compliance with treatment and decreased participation in clinical trials [16].

The Punjab state government has started the Mukh Mantri Punjab Cancer Rahat Kosh Scheme to provide financial support to the patients for cancer treatment [17]. Our results indicated that the patients who are eligible and have applied for health scheme are had a higher chance of completing the treatment as compared to those who are eligible and not applied for the scheme. In low and middle-income countries, most patients did not complete the treatment due to financial barriers. Hence, the elimination of financial barriers plays an important role in the successful completion of the treatment. Furthermore, it is reported that lack of health insurance or inadequate health insurance is a major barrier in seeking preventive services and adequate treatment [18]. In contrast, Sri Lanka provides most cancer treatment free of cost at the National Cancer Institute of Colombo [19].

Age is also an important factor for the completion of the treatment. Maybe due to other comorbidities and social reasons, older age group patients have not completed treatment. The study conducted in a rural hospital of West Bengal has reported that more than 50% of old age (>50 years) cervical cancer patients could not complete the treatment. A study conducted in India has reported that older age (>60 years) are showing less compliance to treatment completion (RT and CT) [20].

The patient who was offered curative treatment had a higher chance of completing the treatment as compared to those who were offered palliative treatment. This finding is consistent with the other hospital-based cancer registries from Mumbai, Bangalore and Dibrugarh [3]. The higher compliance towards the curative treatment might be because patients and their caretakers believe that the chances of cure are better in the curative treatment. Whereas, poor compliance to palliative treatment might be because of fewer hopes for a cure, as the treatment is mainly focused on relieving the pain and improving the quality of life.

The religion has not shown any effect on the completion of the treatment. The study comprises 68% patients from the Sikh religion, 28% Hindu and very few patients from other religions. It is reported that religious fatalism is associated with worse compliance to screening and treatment [21, 22].

In the analysis, we have noted the treatment completion was 30% less in new cases as compared to old cases. This was influenced by the effect of payment mode and intention of the treatment.

It is noted that in localised and loco-regional cases, the treatment completion rate is high when compared to distant metastatic cases. Mumbai, Bangalore and Chennai hospital-based cancer registries also reported that in localised and regional cases, cancer directed treatment is high when compared to the distant stage [3]. In our study, there is no association between clinical extent and treatment completion.

It is observed that in our study, the majority of the cancer patients have undergone the CT and RT treatment. The 5% male and 5% female cases have undergone surgery alone treatment. However, the surgery in combination with RT and CT is 15%. These findings are in comparison with other hospitals in India like Regional Cancer Centre – Thiruvananthapuram (male: 6.5%; female: 7.8%), BBCI (male: 3.3%; female: 5.3%) and Postgraduate Institute of Medical Education and Research (male: 5.7%; female: 7.1%) [3].

In India, treatment-related decisions are generally taken by family members or close relatives. This decision is dependent on the financial burden on the family and easy access for the treatment as well as other social issues. This data is abstracted by the hospital-based registry staff from the EMR. We have not collected the other information which influences the treatment. It is reported that financial, social communication, logistic barrier and medical comorbidities are the major barriers in treatment completion [23].

Providing cancer care services to all is an important component of cancer control. The recent article published by Boyle *et al* [24] has mentioned that to prevent all cancers that can be prevented, treat all cancers that can be treated, cure all cancers that can be cured and provide palliation whenever palliation is required [24]. We need a lot of dedicated efforts at all levels to implement these four pillars of oncology. Cancer is a major public health concern and there is a lack of access to cancer diagnosis and treatment in low and middle-income countries. We need to provide easy access to diagnosis and treatment and provide financial support to the patient through different schemes so that treatment completion will improve. A lot of researchers are focusing on the new interventions to treat cancer effectively. However, along with that, our priority should be in offering the best available treatment to cancer patients to improve the treatment completion which further improves the prognosis and prevent early death. If we prevent early death due to cancer, it has an impact on the economy of the country. It is reported that productivity losses due to premature death from cancer in India is US$ 7.2 Billion and cost per cancer death is US$ 21,096 [25]. The treatment completion has an impact on the deaths of the cancer patients which further effects economy due to lose of person year of life. Hence, if we prevent early death due to cancer, it will have positive impact on health economy.

The limitations of our study are that we did not collect the qualitative data such as family support, patient–doctor communication, fear of treatment, accommodation near the hospital and challenges in receiving financial support from the government schemes. Also, the data abstraction is done from EMRs only.

The reason for noncompliance to treatment depends on the treatment offered. In HBCH, some of the advanced treatment and palliative care facilities are in the development stage. This may be the reason for not completing the treatment by some patients and some patients might have completed the treatment in other centres. It is very challenging to provide the cancer care services in rural set-up. The dedicated team of HBCH Sangrur is playing an important role in providing cancer services to the rural population of Punjab state.

In keeping with the results regarding treatment completion in the present study, the implications for policymakers are as follows: the hospital administration has to address all the challenges faced by the patients and caregivers to increase treatment compliance, such as arrangement of a special desk for patient guidance, improvement in providing health scheme facilities from the government, arrangement of transport services in co-ordination with the local government, arrangement of accommodation for the patient and their caregivers in a dormitory. The emphasis has to be made in assisting the illiterate patients by appointing the social worker/patient guide for all the above support as well as communicating with the treating clinician. In this scenario, the hospital may consider initiating programmes like patient navigation program – KEVAT which was first started in Tata Memorial Hospital to navigate the patient right from the entry in the hospital to follow-up in a holistic way [26]. Furthermore, regular follow-up of the patients who have left the treatment has to be done on priority basis.

## Conclusion

The important findings of this study are that young patients, educated (literate), distance from the hospital staying in Sangrur district (i.e. near to the treating hospital), those who received curative treatment and those who have applied for the government health schemes for financial support have impact on treatment completion. The other variables like the clinical extent of the disease, religion, gender and income have not shown any effect on the treatment completion. This study highlights the social determinants of treatment compliance that has significant effect on outcome of cancer treatment.

As discussed earlier, changes in the hospital system have to be implemented for the improvement of treatment completion. Furthermore, the hospital has to develop the proper referral system to nearby hospital if there is no infrastructure, for example, positron emission tomography scan, treatment for neurology cases, treatment for thoracic and gastrointestinal cancer patient, bone marrow transplantation, etc. There are several reasons for the noncompliance to the treatment. Hence, further studies are required to address this issue. The HBCR is an important source to monitor the cancer care services. This study recommends to raise the awareness among cancer patients about the availability of different state/central government health schemes for financial support.

## List of abbreviations

HBCR, Hospital based cancer registry; HBCH, Homi Bhabha Cancer Hospital; TMC, Tata Memorial Centre, Mumbai, India; CCE, Centre for Cancer Epidemiology; EMR, Electronic medical record; OR, Odds ratio; CI, Confidence interval; CT, Chemotherapy; RT, Radiotherapy.

## Funding sources

The HBCR Sangrur is funded by Tata Memorial Centre, Mumbai, which is a grant in aid institute of the Department of Atomic Energy, Government of India.

## Conflicts of interest

There were no conflicts of interest.

## Authors’ contributions

Atul M Budukh: conceptualisation, methodology, formal analysis, data analysis and writing the original draft. Debashish Chaudhary: treating consultant, data abstraction, review and assistance in writing the draft. Sankalp Sancheti: pathologist, data abstraction, quality control, review and assistance in writing the draft. Tapas Dora: radiotherapist, data abstraction, review and assistance in writing the draft. Alok Kumar Goel: medical oncologist, data abstraction, review and assistance in writing the draft. Anshul Singla: head & neck surgeon, data abstraction, review and assistance in writing the draft. Akash Sali: pathologist, data abstraction, quality control, review and assistance in writing the draft. Shraddha Shinde: case abstraction, data analysis and quality control. Kuldeep Singh Chauhan: case abstraction, data analysis and quality control. Prithviraj Kadam: overall supervision, case abstraction, data analysis and quality control. Raza Mohammad: quality control and statistical data analysis. Rakesh Kapoor: project administration, and assistance in writing the draft. Pankaj Chaturvedi: review of literature, quality control and assistance in writing the draft. Rajesh P Dikshit: review of literature, quality control and assistance in writing the draft. Rajendra A Badwe: overall administration, quality control and technical assistance.

## Figures and Tables

**Table 1.** Socio-demographic characteristics of the cancer patient treated in the HBCH: 2018.

Socio-demographic characteristics	Frequency (*N*)	Percentage (%)
**Total**	**2,528**	**100.0**
Age (in years)		
<40	275	10.9
40–59	1,152	45.6
60+	1,101	43.6
Gender		
Male	1,158	45.8
Female	1,370	54.2
Income (INR per month)		
High (>30,374)	107	4.2
Medium(11,362–30,374)	159	6.3
Low (<11,362)	2,262	89.5
Religion		
Sikh	1,725	68.2
Hindu	710	28.1
Others	93	3.7
Education		
Illiterate	1,403	55.5
Literate	1,125	44.5
District		
Sangrur	944	37.3
Other district	1,584	62.7
State		
Punjab	2,282	90.3
Other state	246	9.7
Type of case		
Old case	515	20.4
New case	2,013	79.6
Clinical extent		
Localisation	554	21.9
Loco-regional	922	36.5
Distant metastasis	704	27.9
Not applicable/unknown	348	13.8
Intent of treatment		
Palliative	536	21.2
Curative	1,186	46.9
Not applicable	806	31.9
Payment mode		
Govt. scheme eligible & applied	1,164	46.0
Govt. scheme eligible & not applied	818	32.4
By own	312	12.3
Govt. employees to file for reimbursement/other health insurance scheme	234	9.3

**Table 2.** Treatment details of the cancer patient treated in the HBCH: 2018.

Treatment	Male		Female		Total	
	Number	%	Number	%	Number	%
Surgery	58	5.0	68	5.0	126	5.0
CT^a^	103	8.9	133	9.7	236	9.3
RT^b^	103	8.9	106	7.7	209	8.3
CT + RT	205	17.7	208	15.2	413	16.3
Surgery + CT	28	2.4	53	3.9	81	3.2
Surgery + RT	58	5.0	47	3.4	105	4.2
Surgery + CT + RT	50	4.3	142	10.4	192	7.6
No treatment	553	47.8	613	44.7	1166	46.1
**Total**	**1,158**	**100.0**	**1,370**	**100.0**	**2528**	**100.0**

a CT, Chemotherapy

b RT, Radiotherapy

**Table 3.** Univariate and multivariate analysis to know the effect of study variables on the treatment completion.

All sites						
Socio-demographic characteristics	Treatment completed *N* (%)	Treatment not completed *N* (%)	Univariate		Multivariate	
			OR (95% CI)	*p-value*	Adjusted OR (95% CI)	*p-value*
Total = 2,528	1,362 (53.9%)	1,166 (46.1%)				
Age (in years)						
<40®	173 (62.9%)	102 (37.1%)	1		1	
40–59	667 (57.9%)	485 (42.1%)	0.81 (0.62–1.06)	0.130	0.63 (0.38–1.03)	0.066
60+	522 (47.4%)	579 (52.6%)	0.53 (0.41–0.70)	0.000	0.52 (0.31–0.86)	0.011
Gender						
Male®	605 (52.3%)	553 (47.7%)	1			
Female	757 (55.3%)	613 (44.7%)	1.13 (0.96–1.32)	0.130		
Income (INR per month)						
High (>30,374)®	53 (49.5%)	54 (50.47%)	1			
Medium (11,362–30,374)	95 (59.8%)	64 (40.2%)	1.51 (0.92–2.48)	0.101		
Low (<11,362)	1,214 (53.7%)	1,048 (46.3%)	1.18 (0.80–1.74)	0.402		
Religion						
Sikh®	916 (53.1%)	809 (46.9%)	1			
Hindu	394 (55.5%)	316 (44.5%)	1.10 (0.92–1.31)	0.282		
Others	52 (55.9%)	41 (44.1%)	1.12 (0.74–1.71)	0.597		
Education						
Illiterate®	715 (51.0%)	688 (49.0%)	1		1	
Literate	647 (57.5%)	478 (42.5%)	1.30 (1.11–1.53)	0.001	1.49 (1.13–1.96)	0.004
District						
Sangrur®	543 (57.5%)	401 (42.5%)	1		1	
Other district	819 (51.7%)	765 (48.3%)	0.79 (0.67–0.93)	0.005	0.67 (0.50–0.89)	0.006
State						
Punjab®	1230 (53.9%)	1052 (46.1%)	1			
Other state	132 (53.7%)	114 (46.3%)	0.99 (0.76–1.29)	0.942		
Type of case						
Old case®	256 (49.7%)	259 (50.3%)	1		1	
New case	1,106 (54.9%)	907 (45.1%)	1.23 (1.02–1.50)	0.034	0.70 (0.50–0.98)	0.037
Clinical extent						
Localisation®	337 (60.8%)	217 (39.2%)	1		1	
Loco-regional	571 (61.9%)	351 (38.1%)	1.05 (0.84–1.30)	0.674	0.89 (0.62–1.27)	0.508
Distant metastasis	357 (50.7%)	347 (49.3%)	0.66 (0.53–0.83)	0.000	1.19 (0.75–1.89)	0.464
Not applicable/unknown	97 (27.9%)	251 (72.1%)	0.25 (0.19–0.33)	0.000	0.91 (0.54–1.58)	0.766
Intent of treatment						
Palliative®	366 (68.3%)	170 (31.7%)	1		1	
Curative	996 (84.0%)	190 (16.0%)	2.43 (1.92–3.09)	0.000	2.70 (1.88–3.88)	0.000
Not applicable	-	806 (100%)	-	-	-	-
Payment mode						
Govt. scheme eligible & applied®	956 (82.1%)	208 (17.9%)	1		1	
Govt. scheme eligible & not applied	138 (16.9%)	680 (83.1%)	0.04 (0.03–0.06)	0.000	0.13 (0.10–0.19)	0.000
By own	149 (47.8%)	163 (52.2%)	0.20 (0.15–0.26)	0.000	0.36 (0.25–0.53)	0.000
Govt. employees to file for reimbursement/other health insurance scheme	119 (50.9%)	115 (49.1%)	0.23 (0.17–0.30)	0.000	0.48 (0.29–0.77)	0.003

®, Reference; *N* (%), Frequency (percentage); CI, Confidence interval
